# Identification and Characterization of a Highly Active Hyaluronan Lyase from *Enterobacter asburiae*

**DOI:** 10.3390/md22090399

**Published:** 2024-08-31

**Authors:** Linjing Zhang, Jiayu Jiang, Wei Liu, Lianlong Wang, Zhiyuan Yao, Heng Li, Jinsong Gong, Chuanli Kang, Lei Liu, Zhenghong Xu, Jinsong Shi

**Affiliations:** 1 Key Laboratory of Carbohydrate Chemistry and Biotechnology, Ministry of Education, School of Life Sciences and Health Engineering, Jiangnan University, Wuxi 214122, China; 2 Shandong Engineering Laboratory of Sodium Hyaluronate and Its Derivatives, Shandong Focusfreda Biotech Co., Ltd., Qufu 273165, China; 3 Innovation Center for Advanced Brewing Science and Technology, College of Biomass Science and Engineering, Sichuan University, Chengdu 610065, China

**Keywords:** hyaluronan lyase, hyaluronic acid, hyaluronic acid oligosaccharides, *Enterobacter asburiae*

## Abstract

Hyaluronic acid (HA) is a well-known functional marine polysaccharide. The utilization and derivative development of HA are of great interest. Hyaluronan lyase has wide application prospects in the production of HA oligosaccharides and lower molecular weight HA. In this study, a strain of *Enterobacter asburiae* CGJ001 with high hyaluronan lyase activity was screened from industrial wastewater. This strain exhibited an impressive enzyme activity of 40,576 U/mL after being incubated for 14 h. Whole genome sequencing analysis revealed that *E. asburiae* CGJ001 contained a cluster of genes involved in HA degradation, transport, and metabolism. A newly identified enzyme responsible for glycosaminoglycan degradation was designated as HylEP0006. A strain of *E. coli* BL21(DE3)/*p*ET-22b(+)*-hylEP0006* was successfully constructed. HylEP0006 exhibited optimal degradation at 40 °C and pH 7.0, showing a high activity of 9.5 × 10^5^ U/mg. HylEP0006 showed specific activity against HA. The minimum degradation fragment of HylEP0006 was hyaluronan tetrasaccharides, and HylEP0006 could efficiently degrade HA into unsaturated disaccharides (HA2), with HA2 as the final product. These characteristics indicate that HylEP0006 has a potential application prospect for the extraction and utilization of hyaluronic acid.

## 1. Introduction

Hyaluronic acid (HA) is a sticky high-molecular-weight polysaccharide, with the unbranched disaccharide of *N*-acetyl glucose and D-glucuronic acid linked through β-1,4 glycoside bond as repeating units connected through β-1,3 glycosidic bonds [1]. The unique properties of HA, including high viscoelasticity and plasticity, exceptional water retention capacity, and permeability, have led to it being extensively utilized in the fields of medicine, clinical diagnosis, cosmetics, and health food industries [2].

Traditionally, hyaluronic acid is mainly obtained from mammalian tissues, including the rooster comb, cartilage, and umbilical cord. Despite the high cost of production, the process is relatively complex, and the large-scale preparation is difficult. The exploration of glycoconjugates originating from microorganisms, especially marine organisms, is gaining increasing attention due to the concern generated by bovine spongiform encephalopathy (BSE) and other food chain crises. Current research focuses on the utilization of inherently secure aquatic organisms as raw materials, aiming to not only promote waste utilization and comprehensive development but also reduce the extraction cost of hyaluronic acid [3]. The rational utilization of marine hyaluronic acid resources can play a comprehensive role in the development and waste utilization effect.

Recent studies have shown that the biological activities of HA are directly related to their relative molecular weight (Mw). In contrast, HA with different molecular weights can exhibit opposite biological activities [4,5]. The HA polymer with higher molecular weight (Mw > 2 × 10^6^ Da) has outstanding properties of elasticity and moisturizing and the function of inflammation inhibition and lubrication, which is commonly used for ophthalmic surgery adhesives and joint injection in ophthalmic surgery [6]. The HA with Mw of (1–2) × 10^6^ Da exerts preferable lubrication, moisturizing, and drug slow-release properties, which can be applied in cosmetics, eye drops, skin burn healing, and postoperative anti-adhesion [7]. The HA with lower molecular weight (Mw ≤ 1 × 10^4^ Da) (including Hyaluronan oligosaccharides, o-HAs) has demonstrated immunological activities of activating endothelial cells and inhibiting multidrug resistance in tumors [8,9], which could be broadly applied in medicine. Comparatively, HA with lower molecular weight can maintain its activities while being readily absorbed by the human body [10]. Consequently, research on HA with lower molecular weight has been a focal point [11].

At present, HA with lower molecular weight is mainly obtained through the degradation of HA with high molecular weight by physical (ultrasonication, microwave irradiation, and heating), chemical (acidic and alkaline hydrolysis, oxidant hydrolysis), and enzymatic methods. The resultant products employing either physical or chemical methodologies usually comprise a composite of polydisperse oligosaccharides and monosaccharides [12], complicating subsequent purification processes and making it challenging to obtain HA oligosaccharides with particular molecular weight. Moreover, HA oligosaccharides can also be synthesized from its monosaccharide precursor through chemical methods. However, time-consuming reaction processes, rare carbohydrate oligosaccharide backbones, and expensive substrates (uridine diphosphate (UDP)-sugars) obstruct the large-scale production of HA oligosaccharides [13]. In contrast, the utilization of well-characterized hyaluronidase (HAase) enzymes for producing HA oligosaccharides presents a highly promising and appealing alternative, owing to its distinctive advantages of mild operating conditions, high degradation rates, and high product homogeneity [14]. Microbial-derived hyaluronidase is plentiful, easy to recombinant expression, convenient for extraction, and relatively simple for high purification. Therefore, it is the primary source of enzyme for application. Since the significance of bacterial hyaluronidase, various microorganisms have been explored to produce hyaluronidase. Recently, it was reported that *Bacillus* sp. A50 produced hyaluronic acid lyase from the deep sea [15], and a hyaluronic acid lyase (BniHL) was purified from *Bacillus niacin* strain JAM F8 [16]. A hyaluronate lyase was found in *Haliscomenobacter hydrossis* from active sludge, which was rich in the microorganism community, playing an important role in treating public wastewater [17]. However, current progress in lower enzyme activity, unclear degradation products, and low yield limit the production of hyaluronidase and industrial production of oligosaccharides by enzymatic methods. Consequently, it is necessary to obtain better microbial hyaluronidase and clarify the characteristics for HA industry promotion.

Therefore, this study aims to screen and isolate microorganism strains harboring high-activity hyaluronan lyase and characterize the enzymatic properties of the obtained hyaluronan lyase to supplement the enzyme resources further.

## 2. Results

### 2.1. Isolation and Identification of Enterobacter sp. CGJ001

**Figure 1:**
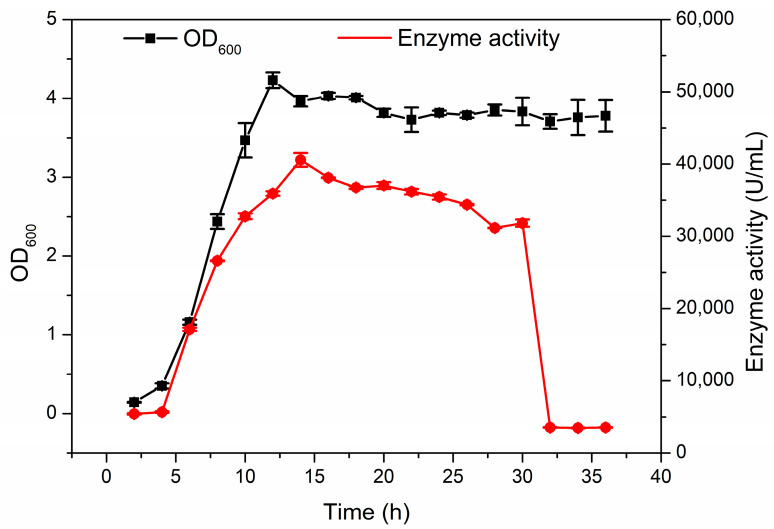
Typical time course of the growth of *Enterobacter* sp. CGJ001 in a shake flask. Enzyme activity (red, U/mL) and cell growth density (black, OD_600_ values) were measured regularly. Values represent the mean of three replicates ± SD.

The strain *Enterobacter* sp. CGJ001 was successfully screened from industrial wastewater, which can grow well in culture with the single carbon source of hyaluronic acid. The strain of *Enterobacter* sp. CGJ001 was cultured by fermentation and reached a stable growth phase at 12 h. A large amount of hyaluronic acid lyase (intracellular enzyme activity) began to be produced around 6 h and reached the highest level of enzyme activity, 40,576 U/mL at 14 h, as shown in Figure 1.

**Figure 2:**
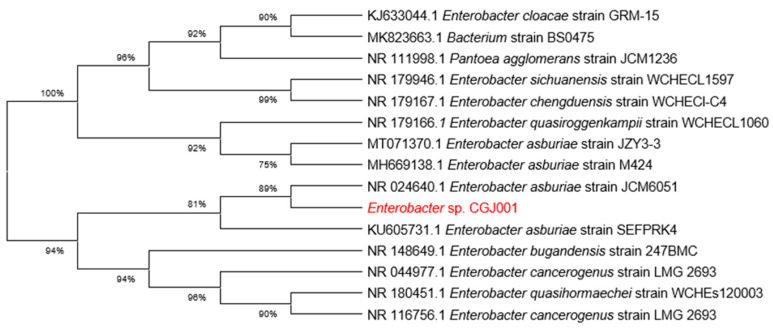
The phylogenetic tree of strain *Enterobacter* sp. CGJ001 was constructed based on the analysis of 16S rDNA sequences, and the phylogenetic tree was generated by MEGA X using the neighbor–joining method.

The length of the 16S rDNA sequence from strain *Enterobacter* sp. CGJ001 was found to be 1381 bp. A sequence blast alignment was conducted using the NCBI database to investigate its phylogenetic relationship, and a set of 14 type strains were chosen for further analysis. The phylogenetic tree analysis revealed that strain *Enterobacter* sp. CGJ001 exhibited the highest similarity of 99.85% with *Enterobacter asburiae* JCM6051 (NR 024640.1), as depicted in Figure 2. Consequently, strain *Enterobacter* sp. CGJ001 was designated as *Enterobacter asburiae* CGJ001.

### 2.2. Whole-Genome Analysis of E. asburiae CGJ001 and Prediction of PUL_HA_

**Figure 3:**
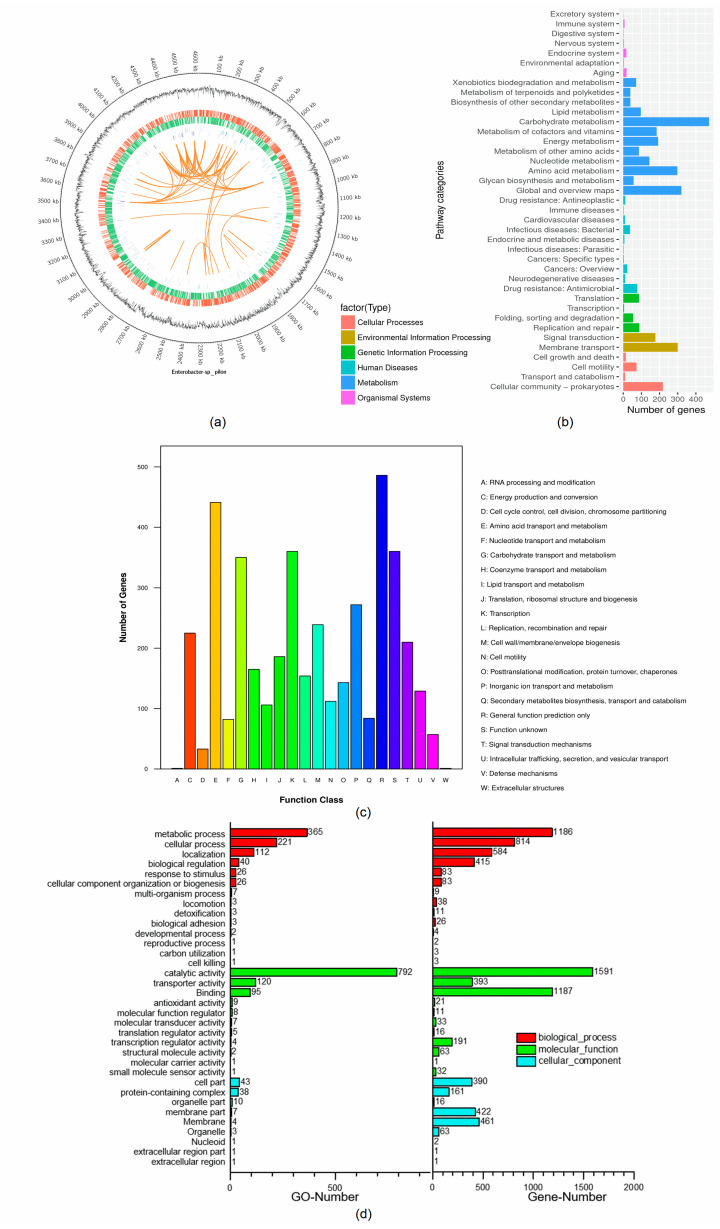
Whole-genome analysis of *E. asburiae* CGJ001: (**a**) Chromosome genome pattern of *E. asburiae* CGJ001. The circle diagram shows seven kinds of information, from outside to inside: The first circle is the genomic position information, the second circle is the GC content information, the third circle is the coding genes on the plus strand (red marks), the fourth circle is the coding genes on the minus strand (green marks), the fifth circle is the ncRNA information on the plus strand (blue marks), and the sixth circle is the ncRNA information on the minus strand (purple marks). The seventh circle is marked with information on long genomic repeats (orange marks). (**b**) KEGG metabolic pathway secondary classification *of E. asburiae* CGJ001. KEGG classified the biological metabolic pathways into 6 categories, and each category was systematically divided into secondary classifications. The number of genes in each metabolic pathway in the secondary classification was counted. (**c**) COG functional classification statistics *of E. asburiae* CGJ001. (**d**) GO functional classification statistics *of E. asburiae* CGJ001.

Whole-genome sequencing analysis showed that the complete genome of *E. asburiae* CGJ001 was composed of the main chromosome of 4,610,415 bp and one plasmid, which was named plasmid A (265,602 bp), with an overall GC content of 57.18%. Its chromosomes contained 4425 total genes, 82 tRNAs, 25 rRNAs, and 200 tandem repeats, as shown in Figure 3a and Table 1. The 4193 coding genes in the whole genome of *E. asburiae* CGJ001 were compared with the database to obtain the functional KEGG, GO, and COG annotation information. A total of 2991 genes were functionally annotated in the KEGG database, as shown in Figure 3b, of which 1667 genes were annotated in various metabolic pathways, and mainly distributed in metabolic pathways and environmental information processing pathways. The most number of genes in the metabolic pathway was located in carbohydrate metabolism (472 genes). The annotation of 472 genes indicated *E. asburiae* CGJ001 had intense functions of carbohydrate catabolism and metabolism. As shown in Figure 3c, COG database annotation results showed that functional proteins related to the transport and metabolism of carbohydrate substances and amino acids were highly annotated, indicating that this strain had a strong ability for carbohydrate metabolism. In addition, Figure 3d showed that 3629 genes could be annotated in GO database with three kinds of function. Among the molecular functions, the top annotated function in the number of genes was catalysis (792), followed by binding and transport. Genes annotated to the biological process also count high, with metabolic and cellular process as the two main classifications. Those genes related to cell components were mainly annotated in cells, cell components, and organelles.

### 2.3. Prediction of PUL_HA_

**Figure 4:**
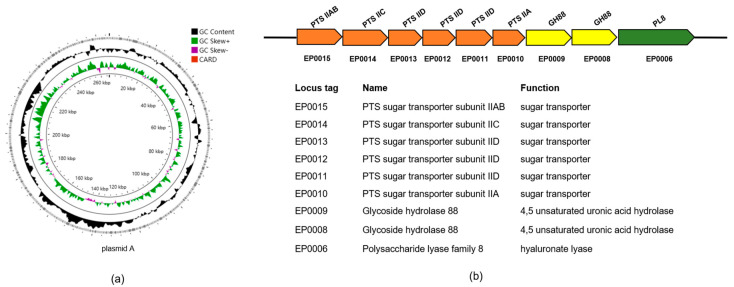
The analysis of plasmid A: (**a**) Plasmid A genome pattern of *E. asburiae* CGJ001. (**b**) The PUL_HA_ was anticipated in plasmid A of strain *E. asburiae* CGJ001.

The genes associated with HA utilization in strain *E. asburiae* CGJ001 clustered in a genomic region of plasmid A, as shown in Figure 4a, suggesting that this gene cluster might be the polysaccharide utilization site of HA (PUL_HA_). The PUL_HA_ in the genome consisted of six components: PL8 family hyaluronan lyase (EP0006), two GH88 family unsaturated glucuronate hydrolases, and a sugar transporter glucose phosphotransferase system (PTS) (Figure 4b). Since this enzyme is an intracellular enzyme, it is hypothesized that hyaluronan lyase PL18 is embedded on the cell surface and degrades hyaluronan to unsaturated disaccharides. Unsaturated disaccharides are transported to the cytoplasmic periphery by the TonB transport system and then imported into the cytoplasm by the sugar transporter glucose phosphorylase system PTS. The degradation process facilitated by the unsaturated glucuronate hydrolase GH88 undergoes within the cytoplasm, forming 4,5 unsaturated glucuronic acid (ΔGlcUA) and *N*-acetyl D-glucosamine (GlcNAc) through the cleavage of β-1,4 bonds. ΔGlcUA and GlcNAc are subsequently involved in further metabolism processes.

The Hyaluronan Lyase (*hylEP0006*) gene has a length of 2397 bp and encodes a protein consisting of 799 amino acid residues. The predicted Mw and isoelectric point (pI) values of HylEP0006 are 87.93 kDa and 8.68, respectively. By utilizing SignalP-5.0, we identified a type I signal peptide at the N-terminus of HylEP0006 composed of 20 amino acid residues (MKKTNLAFSLLCLSMGSVHA). The CD search results show that HylEP0006 contains a GAG_lyase superfamily module (Ile^46^–Pro^745^). By the amino acid alignment, HylEP0006 could be attributed to the PL8 family, which included the conserved catalytic residues of the PL8 family with Asn^234^, His^284^, and Tyr^293^.

### 2.4. Heterogenous Expression of hylEP0006

**Figure 5:**
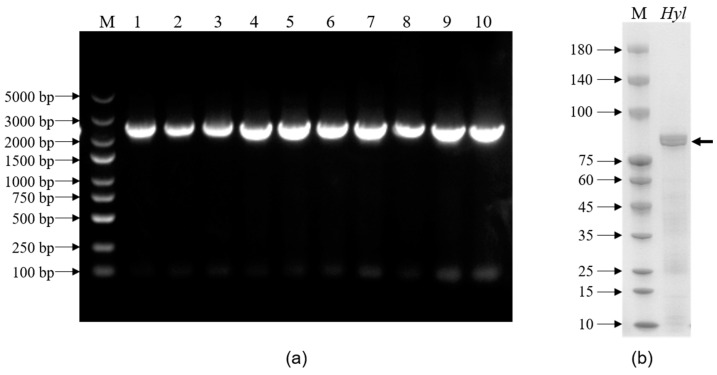
The expression of *hylEP0006*: (**a**) Colony PCR validation of the recombinant strain. Lane M: DNA marker; 1–10: PCR amplification bands of positive clones (the DNA fragment size of *hylEP0006*: 2379 bp). (**b**) SDS-PAGE of purified recombinant HylEP0006. Lane M, unstained protein molecular weight marker; lane *Hyl*, purified HylEP0006.

As depicted in Figure 5a, the strain of *E. coli* BL21(DE3)/*p*ET-22b(+)-*hylEP0006* was successfully constructed. Subsequently, purification was successfully accomplished using affinity chromatography. In Figure 5b, a distinct band within the 75–100 kDa was observed on SDS-PAGE analysis, aligning with the anticipated size of HylEP0006 (87.88 kDa). The protein concentration measured by the Bradford method was 1.15 mg/mL, the enzyme activity was 1.1 × 10^6^ U/mL, and the specific enzyme activity of hyaluronan lyase HylEP0006 was 9.5 × 10^5^ U/mg.

### 2.5. Biochemical Properties Characterization of HylEP0006

#### 2.5.1. The Effects of Temperature and pH on Enzymatic Activity

**Figure 6:**
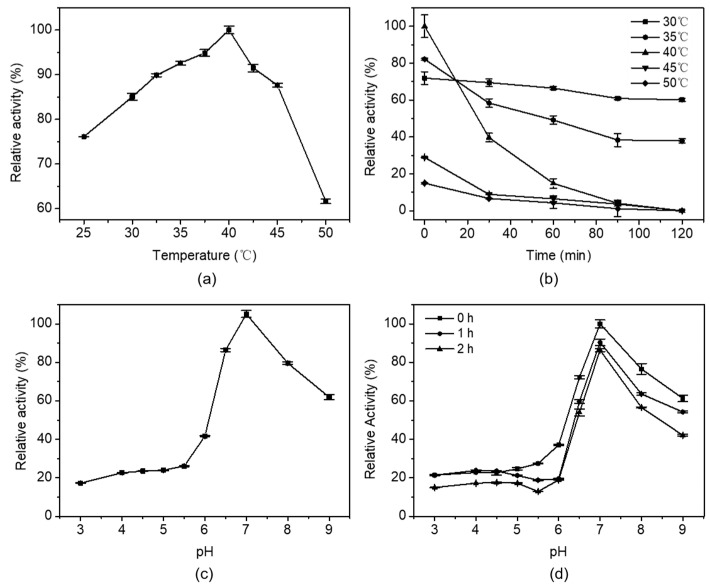
The effects of temperature and pH on the enzymatic activity of HylEP0006: (**a**) Effect of temperature. The enzymatic activity of HylEP0006 was measured at 25–50 °C. HylEP0006 had the highest specific activity at 40 °C, equivalent to 100%. (**b**) Thermal stability of HylEP0006. The cells were incubated at different temperatures (30–50 °C) for 2 h, and the residual activity of HylEP0006 was determined at 40 °C. The initial specific activities of HylEP0006 were all set to be 100%. (**c**) Effect of pH. The enzymatic activity of HylEP0006 was assessed in a 50 mM buffer solution, comprising Na_2_HPO_4_-Citrate buffer (pH 3.0–5.0), NaH_2_PO_4_-Na_2_HPO_4_ buffer (pH 6.0–8.0), and Glycine-NaOH buffer (pH 9.0–10.0). In NaH_2_PO_4_-Na_2_HPO_4_ buffer (pH 7.0), HylEP0006 exhibited its maximum specific activity, which was recorded as being equivalent to 100%. (**d**) pH stability of HylEP0006. The residual activity of HylEP0006 was assessed at a temperature of 40 °C by subjecting it to incubation in the aforementioned buffer (with pH ranging from 3.0 to 9.0) for 2 h, maintaining the incubation temperature at 30 °C. The initial specific activities of HylEP0006 were all set to be 100%. Values represent the mean of three replicates ± SD.

The recombinant HylEP0006 exhibited maximal activity at 40 °C, as shown in Figure 6a. The stability of HylEP0006 was better at 30–35 °C, and the activity of HylEP0006 could retain about 60% when it was kept for 2 h, as shown in Figure 6b. The stability of the enzyme was poor at the temperature of 40 °C and above, and the activity rapidly decreased to less than 50% after 30 min of heat preservation.

Figure 6c indicated that HylEP0006 was a neutral hyaluronan lyase, and the optimum pH of this enzyme was determined as pH 7.0. When the pH value was between 5.0 and 9.0, the activity was relatively stable, as shown in Figure 6d.

#### 2.5.2. Substrate Specificity of HylEP0006

The specificity of hyaluronan lyase against different substances is also different. Table 2 showed that the HylEP0006 performed good substrate specificity and high substrate specificity for hyaluronic acid, but there was almost no degradation for chondroitin sulfate, heparin, sodium alginate, and chitosan.

#### 2.5.3. Effects of Metal Ions on HylEP0006

As shown in Table 3, when the concentration of metal ions was 1 mmol/L, Ca^2+^ can promote the activity of HylEP0006, while Al^3+^, Cu^2+^, Zn^2+^, and Fe^3+^ could significantly inhibit the activity of HylEP0006. When the concentration of metal ions was increased to 10 mmol/L, both Mg^2+^ and Ca^2+^ could promote the activity of HylEP0006. With the increase in the concentration of the two metal ions, the promoting effect was more obvious, while the rest of the metal ions showed an inhibitory trend on the activity of HylEP0006.

#### 2.5.4. Kinetic Constants of HylEP0006

**Figure 7:**
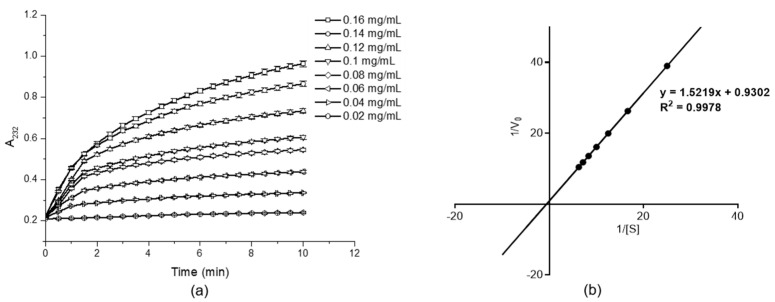
Enzymatic kinetic study of HylEP0006: (**a**) HylEP0006 degradation process of different concentrations of substrate figure. Values represent the mean of three replicates ± SD. (**b**) Lineweaver–Burk double reciprocal plot of HylEP0006.

Figure 7a shows the degradation of substrates at various concentrations by the HylEP0006 at 40 °C, pH 7.0. The highest concentration of HA, which was 0.16 mg/mL, was chosen to determine kinetic parameters. The affinity of HylEP0006 for HA was studied by measuring the kinetic parameters of HA at different concentrations (0.01–0.16 mg/mL). Kinetic parameters were calculated using a Linewever–Burk plot, as shown in Figure 7b. The maximum reaction rate of HylEP0006 was *V*_max_ = 1.075 A_232_ /min, and Michaelstrom constant *K*_m_ = 1.636 mg/mL.

### 2.6. Analysis of Final Degradation Product

**Figure 8:**
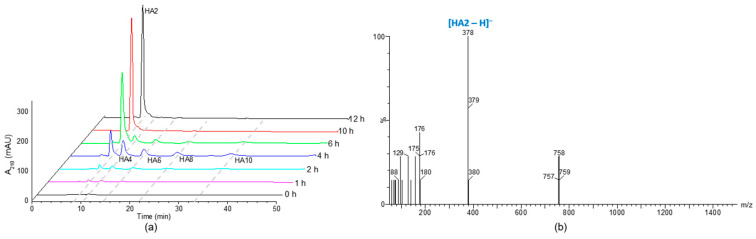
The analysis of HA digested by HylEP0006: (**a**) The final product of HA digested with HylEP0006 at 40 °C was analyzed by HPLC using a YMC-Pack Polyamine II column. (**b**) Analysis of the end product resulting from the digestion of HA with HylEP0006 using electrospray ionization mass spectrometry (ESI-MS).

To investigate the final degradation product of HylEP0006, hyaluronic acid with a molecular weight of 1.3 × 10^7^ Da was subjected to enzymatic degradation. After being degraded for 1 h, the results of HPLC analysis revealed the presence of unsaturated disaccharides (HA2), hyaluronan tetrasaccharide (HA4), hyaluronan hexose (HA6), hyaluronan octadose (HA8), and hyaluronan decasaccharide (HA10), as shown in Figure 8a. For a total degradation of hyaluronic acid for 10 h, it showed that the end product of the enzyme was HA2. To accurately determine the precise molecular weight of the end product, we employed negative ion ESI-MS. The dominant peak observed in the mass spectrum exhibited a *m*/*z* value of 378, which corresponds to the molecular weight of the unsaturated disaccharide [18], as depicted in Figure 8b. Thus, it could be concluded that HylEP0006 eventually degrades HA to unsaturated disaccharides.

### 2.7. Exploration of the Degradation Behavior of HylEP0006

**Figure 9:**
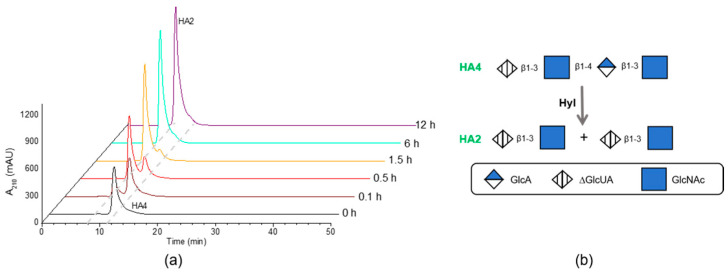
The analysis of HA4 degraded products by HylEP0006: (**a**) The product of HA4 degraded with HylEP0006 at 40 °C was analyzed by HPLC. (**b**) Scheme diagram of the cleavage of oligosaccharide HA4 substrates by HylEP0006.

To further explore the degradation behavior of enzymes, degradation experiments of purified HA4 (Figure S1) and HA10 (Figure S2) with HylEP0006 were investigated. Figure 9a shows that HA2 was generated from HA4 at 0.1 h, and no other product was generated. HA4 could be utterly degraded to HA2 at 6 h. These results indicated that the minimum degradation substrate of HylEP0006 was HA4, and the final degradation product was HA2 (Figure 9b).

**Figure 10:**
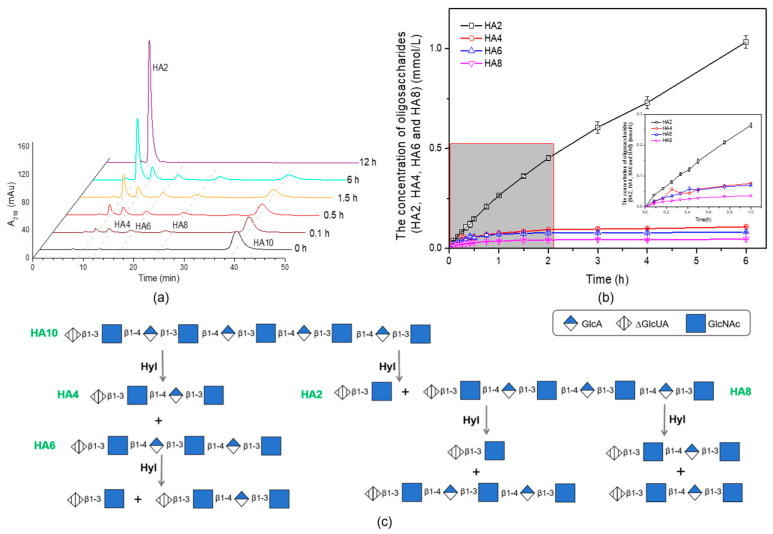
The analysis of HA10 degraded products by HylEP0006: (**a**) The product of HA10 degraded with HylEP0006 at 40 °C was analyzed by HPLC using a YMC-Pack Polyamine II column. (**b**) The oligosaccharide content of each product component during the initial 6 h degradation period of HA10 at 40 °C was analyzed by HPLC. Values represent the mean of three replicates ± SD. (**c**) Scheme diagram of the cleavage of oligosaccharide HA10 substrates by HylEP0006.

As illustrated in Figure 10a, during the degradation process of HA10, the formation of HA2, HA4, HA6, and HA8 could be seen in HPLC profiles at 0.1 h. In detail, at 0.1 h, the concentration of HA2 was 0.0386 mmol/L, while the concentrations of HA4, HA6, and HA8 were 0.0177 mmol/L, 0.0195 mmol/L, and 0.0112 mmol/L, respectively, as showed in Figure 10b. Moreover, during the subsequent degradation process, the concentration of HA4, HA6, and HA8 only increased slowly during the initial 1.5 h degradation period. In contrast, the concentration of HA2 increased steadily with the increase in degradation time. At the end of the degradation, HA10 was eventually degraded to HA2 at 12 h.

## 3. Discussion

HA can be found in numerous functional marine resources, such as discarded fish eyes, loach mucus, stingray liver, and so on. For HA degradation, enzymatic processing employing hyaluronidase is highly efficient and specific, and it can be used as a potential process. Compared with animal-derived enzymes, microbial-derived enzymes have the characteristics of high yield, strong activity, diverse properties, and easy heterologous recombinant expression, which are the main research objects of industrial enzymes. In this study, a strain of *E. asburiae* CGJ001 with high hyaluronic acid degradation ability was screened from industrial wastewater. Reinforced by integrating whole-genome sequencing, we successfully acquired the comprehensive amino acid sequence and the gene encoding the hyaluronate lyase of HylEP0006. Based on bioinformatics analysis, a hyaluronic acid degradation system of PUL_HA_ in *E. asburiae* CGJ001 was identified. This PUL_HA_ was very similar to those PULs of HA in *V. alginolyticus* [18] and *Fusobacteria* [19]. In its genome, *E. asburiae* CGJ001 possesses TBDT proteins, which are the *Proteobacteria* equivalent of the SusC/SusD pair [20]. Combining genomic investigations with biochemical analysis enhanced our comprehension of PULs’ role in microbial communities.

In this study, we conducted heterologous expression, purification, and characterization of hyaluronan lyase in PUL_HA_. The strain of *E. coli* BL21(DE3)/*p*ET-22b(+)*-hylEP0006* was successfully constructed, and HylEP006 exhibited optimal degradation, showing a high activity of 9.5 × 10^5^ U/mg. The activity of HylEP0006 is higher compared to that of mammalian hyaluronidase, including *Homo sapiens*, *Bos grunniens,* and *Lachesis mutar hombeata,* as shown in Table 4. The enzyme can rapidly degrade HA and obtain HA oligosaccharides with concentrated molecular weight. The enzymatic activity of HylEP0006 was optimal at 40 °C and pH 7.0. The activity of HylEP0006 showed the same optimum temperature as that of *Bacillus* sp. CQMU-D, *Paenibacillus aquistagni* SH-7-A, *Yersinia* sp. 298, and *Escherichia* sp. A9, as shown in Table 4. Most bacterial hyaluronan lyases play their most important role in the neutral and acidic range. HylEP0006 exhibits stability at pH 6.0–9.0 and 30–35 °C, comparable to the human environment. This suggests that the HylEP0006 has great potential applications in the medical industry. Moreover, HylEP0006 is stable in a wider pH range, which is beneficial to the storage of enzyme preparations. When the concentration of metal ions increased from 1 to 10 mmol/L, Mg^2+^ and Ca^2+^ promoted the enzymatic activity of HylEP0006, while Al^3+^, Zn^2+^, and Fe^3+^ significantly inhibited the activity. Similar to *Brevibacterium halotolerans* DC1, Ca^2+^ and Mg^2+^ promote hyaluronan lyase activity, whereas Zn^2+^ inhibits hyaluronan lyase activity. HA lyases from different sources has different substrate specificities, as shown in Table 4. HylEP0006 possesses high specificity for HA and almost no ability to degrade chondroitin sulfate and heparin. Due to its specific ability to degrade HA, HylEP0006 holds great potential in extracting and utilizing marine HA resources.

HylEP0006 was used to degrade the hyaluronan polysaccharide, which was completely degraded at 12 h of degradation, and the product was a singly unsaturated hyaluronan disaccharide. Like most hyaluronan lyases, the final degradation product is an unsaturated hyaluronan disaccharide. The degradation mode of HylEP0006 was further explored. The purified enzyme efficiently degraded a singular substrate (HA4), resulting in complete degradation to HA2 within 6 h, as shown in Figure 9a. This indicated that the minimal binding substrate of the enzyme was unsaturated hyaluronan tetrasaccharide, and a schematic daigram of the degradation is shown in Figure 9b. Degradation of the single substrate HA10 with purified enzymes resulted in the formation of HA2. Similarly, after 12 h, all HA10 was degraded to HA2 (Figure 10a). As illustrated in Figure 10b, HA2, HA4, HA6, and HA8 were all generated at 0.1 h, indicating that HA2 was not the only product generated by HylEP0006. The concentrations of HA4 and HA6 fluctuated at 0.3 h and 0.5 h, respectively, which could be attributed to the fact that the amounts of HA4 and HA6 being degraded by HylEP0006 at that time were higher than those produced. In addition, the concentrations of HA4, HA6, and HA8 increased slowly during the initial 1.5 h degradation period, while the concentration of HA2 increased steadily with the increase in degradation time. Therefore, it indicated that HylEP0006 exhibited a higher propensity for generating unsaturated hyaluronic acid disaccharides during the degradation of HA. The mechanism diagram of HylEP0006 in the degradation of HA is shown in Figure 10c. HylEP0006 not only degrades HA in a short time but also obtains relatively pure unsaturated HA2. The excavation of HylEP0006 supplements the resources of hyaluronate lyase. It could be beneficial to further studies of activity assessments of HA oligosaccharides.

## 4. Materials and Methods

### 4.1. Materials

HA (1.3 × 10^7^ Da) was obtained from Focus Freda (Jinan, China). Restriction endonucleases were purchased from Takara, and DNA polymerases, DNA purification kit, gel extraction kit, and plasmid miniprep kit were obtained from Vazyme (Nanjing, China). All other reagents were purchased from commercial sources.

### 4.2. Methods

#### 4.2.1. Isolation of Hyaluronate Lyase-Producing Bacteria

Samples were obtained from waste water near factories producing HA, and 1 mL of the supernatant was added to 9 mL of normal saline and diluted to five concentration gradients of 10^−4^, 10^−5^, 10^−6^, 10^−7^, and 10^−8^, respectively. The diluted bacterial suspension was spread on the primary screening medium. Two parallel cultures were made for each concentration and incubated at 30 °C for 5 days. Well-grown single colonies were picked out, through liquid medium cultivation, coated in solid medium, and then picked out in seed liquid medium, cultured for 24 h at 30 °C, 220 rpm. The cells were collected by centrifugation, resuspended in NaH_2_PO_4_-Na_2_HPO_4_ buffer (pH 7.0), sonicated and centrifuged to obtain the crude enzyme solution. The enzyme activity was determined, and the strain with high enzyme activity was screened out.

#### 4.2.2. Enzyme Activity Assays

The activity of hyaluronate lyase was measured as described above. Briefly, 0.9 mL, 2 g/L HA (dissolved in 50 mM, pH 7.0 NaH_2_PO_4_-Na_2_HPO_4_ buffer) was reacted with 0.1 mL of the enzyme solution. The released reducing sugar was determined by the 3, 5-dinitrosalicylic acid (DNS) assay, and the absorbance at 540 nm was measured. One unit of the activity of hyaluronate lyase was defined as the amount of enzyme required to release 1.0 μg of reducing sugar, equivalent to glucose, per hour after hydrolysis of HA at the optimal reaction temperature [33].

#### 4.2.3. Identification of Strain *Enterobacter* sp. CGJ001

The forward primer 27f: 5′-AGTTTGATCCTGGCTCAG-3′ and the reverse primer 1492r: 5′-GCTTACCTTGTTACGACTT-3′ were used for 16S rDNA gene amplification of strains. The program used for strain gene amplification consists of pre-denaturation at 95 °C for 10 min, denaturation at 95 °C for 30 s, annealing at 55 °C for 30 s, extension at 72 °C for 90 s, a total of 34 cycles, extension at 72 °C for 15 min, and insulation at 4 °C. By blast in the NCBI database for sequence alignment (http://blast.ncbi.nlm.nih.gov/Blast.cgi, accessed on 5 April 2022), the phylogenetic tree was generated by MEGA X using the neighbor-joining method [34].

#### 4.2.4. Genome Sequencing and Sequence Analysis

Strain *E. asburiae* CGJ001 was fermented in a culture medium for 24 h at a temperature of 30 °C. Following this, the cells were collected by centrifugation at 12,000 rpm at 4 °C for 10 min. Subsequently, the cell collection was sent to Suzhou Golden Extreme Intelligence Technology Co., Ltd. (Suzhou, China), to extract genomic DNA. The whole genome of the strain was sequenced. Based on the assembly results, the genome components, such as functional genes, non-coding RNA, and repetitive sequences, were analyzed. For the predicted genes, the sequence similarity was compared with the relevant database (KEGG, COG, and GO) to obtain the gene function [35].

#### 4.2.5. Sequence Analysis of HylEP0006

The NCBI PDB and Nr databases were searched for similarity using the online Blastp algorithm. Protein modules and domains were analyzed using conserved domain (CD) search technology. The presence and pattern of signal peptides were identified using the SignalP 5.0 server (http://www.cbs.dtu.dk/services/SignalP, accessed on 5 May 2023). The ProtParam tool on the ExPASy server (http://www.expasy.org, accessed on 15 May 2023) was utilized to predict the physicochemical characteristics of proteins, including Mw and pI. 

#### 4.2.6. Molecular Cloning, Protein Expression, and Purification

Using the primers of *hylEP0006*-F (5′-aattaattcggatccgaattcATGGATCGGATCGATATAAGCAC-3′) and primer *hylEP0006*-R (5′-ctcgagtgcggccgcaagcttTTATTCATTGGCAGGTAGCTGATAG-3′), *hylEP0006* was cloned to its full-length gene. The gene fragment was linked between *Eco*R I and *Hin*d III sites of *p*ET-22b(+). The recombinant plasmid *p*ET-22b(+)-*hylEP0006* was transformed into *E. coli* BL21(DE3) cells. The *p*ET-22b(+)-*hylEP0006* was sequenced at Tianlin Biological Co., Ltd., Shanghai, China. Recombinant bacteria cultivation in Luria–Bertani (LB) medium 37 °C, when OD_600_ reached 0.4, 0.02 mM isopropyl β-D-thiogalactoside (IPTG) was added in, and *E. coli* BL21(DE3)/*p*ET-22b(+)-*hylEP0006* was induced at 20 °C for 24 h. The cells were collected by centrifugation, resuspended in NaH_2_PO_4_-Na_2_HPO_4_ buffer (pH 7.0), sonicated and centrifuged to obtain the crude enzyme solution. The recombinant hyaluronate lyase containing C-terminal (His)_6_ tags was purified using Histrap column (Beyotime Biotechnology, Shanghai, China) chromatography from the supernatant solution. The enzyme was collected for characterization, and its purity was verified by sodium dodecyl sulfate-polyacrylamide gel electrophoresis (SDS-PAGE). Protein concentration was determined using the BCA Protein Assay kit (Beyotime Biotechnology, Shanghai, China).

#### 4.2.7. Characterization of HylEP0006

HylEP0006 and HA were incubated at different temperatures (25, 30, 32.5, 35, 37.5, 40, 42.5, 45, and 50 °C) to determine the optimum temperature of HylEP0006. The enzyme was kept at different temperatures (30, 35, 40, 45, and 50 °C) for 2 h, and the enzyme activity was measured every 30 min to investigate the thermal stability of HylEP0006. HylEP0006 and HA were incubated at different pH, including Na_2_HPO_4_-Citrate buffer (pH 3.0–5.0), NaH_2_PO_4_-Na_2_HPO_4_ buffer (pH 6.0–8.0), and Glycine-NaOH buffer (pH 9.0–10.0), to determine the optimal reaction pH of HylEP0006. The enzyme was incubated in buffer with different pH, including Na_2_HPO_4_-Citrate buffer (pH 3.0–5.0), NaH_2_PO_4_-Na_2_HPO_4_ buffer (pH 6.0–8.0), and Glycine-NaOH buffer (pH 9.0–10.0), for 2 h, and the enzyme activity was measured every 1 h to explore the stability of HylEP0006 in different pH buffers. The substrate of the enzyme was changed, and HylEP0006 was used to degrade 2 g/L HA, chondroitin sulfate, heparin, sodium alginate, and chitosan, respectively. The activity of HylEP0006 for degrading HA was used as a control of 100%; the relative enzyme activities of the experimental groups were calculated. To explore the effect of metal ions on hyaluronidase, various metal ion solutions were prepared at the concentrations of 1 mM and 10 mM, respectively (Al^3+^, Mg^2+^, Cu^2+^, Zn^2+^, Fe^3+^, Ba^2+^, Li^+^, K^+^, Ca^2+^). The enzyme activity was determined after being incubated at 30 °C for 30 min in different ion solutions, with the activity in NaH_2_PO_4_-Na_2_HPO_4_ buffer as the control of 100%. The relative enzyme activities of the experimental groups were calculated.

#### 4.2.8. Analysis of Kinetic Parameters

Different concentrations of HA solutions (0.02, 0.04, 0.06, 0.08, 0.10, 0.12, 0.14, and 0.16 g/L) were prepared as the reaction substrate, and the activity of HylEP0006 was determined under the optimal conditions. The enzymatic kinetic parameters of HylEP0006 were calculated using nonlinear analysis with GraphPad Prism 8.0 (GraphPad Software, Inc., San Diego, CA, USA). 

#### 4.2.9. Analysis of Degradation Products of HylEP0006

HPLC was employed for product analysis. The detailed method included: YMC-Pack Polyamine chromatographic column II (250 mm × 4.6 mm, 5 μm), mobile phase: 0.1 mol/L NaH_2_PO_4_ solution: Acetonitrile (*v*/*v* = 90:10), UV detection at 210 nm, column temperature at 30 °C, flow rate at 0.5 mL/min, and loading volume at 5 μL. The negative ion electrospray ionization mass spectrometry (ESI-MS, MALDI SYNAPT MS, Hilo, HI, USA) was utilized to determine the Mw of the end product accurately. The mass spectrum collection range: 50–2000 *m*/*z*. The ESI-MS analysis was performed under the following conditions: Capillary: 3.0 kV, Cone: 20/50 V, Source Block Temp: 100 °C, Desolvation Temp: 400 °C, Desolvation Gas Flow: 700 L/h, Cone Gas Flow: 50 L/h, Collision Energy: 6 eV, Detector: 1800 V [36].

#### 4.2.10. The Degradation Behavior Exploration of HylEP0006

The degradation behavior of HylEP0006 towards HA was studied. For the enzymatic reaction, the ratio of enzyme and substrate was unified as 10 U/mg. A 20 mL solution of HA (0.1%, *w*/*v*) was digested with purified HylEP0006 for 12 h at 40 °C. The samples were taken at incubation times of 1, 2, 4, 6, 10 and 12 h. For further exploration of the reaction detail, HA4 and HA10 were separated and employed as the substrates. The samples were taken at 0.1, 0.3, 0.5, 1, 1.5, 6, and 12 h. All of the samples were subjected to thermal inactivation at 100 °C for 10 min, followed by 12,000 rpm for 10 min. Subsequently, filtration was performed using a cellulose acetate membrane (0.22 μm) for HPLC analysis.

#### 4.2.11. Statistical Analysis

All experiments were conducted in triplicate, and the results are presented in tables and graphs as mean values accompanied by their corresponding standard deviations.

## 5. Conclusions

In this study, a high hyaluronan lyase-producing strain, *E. asburiae* CGJ001, was screened from wastewater. In conjunction with the analysis of whole-genome sequencing, it was discovered that *E. asburiae* CGJ001 harbors a cluster of genes related to the degradation, transportation, and metablism of HA. The strain of *E. coli* BL21(DE3)/*p*ET-22b(+)*-hylEP0006* was successfully constructed, and HylEP0006 exhibited optimal degradation at 40 °C and pH 7.0, showing a high activity of 9.50 × 10^5^ U/mg. The minimal binding substrate of HylEP0006 was an unsaturated tetrasaccharide of hyaluronic acid, and HylEP0006 efficiently prepared unsaturated disaccharides. This research aims to enhance the utilization potentiality of hyaluronan lyase across various industries, including medicine, cosmetics, and food sectors. In addition, the extraction and utilization of marine hyaluronic acid resources are promoted through microbial-derived enzymes.

## Figures and Tables

**Table 1:** *E. asburiae* CGJ001 genome basic information.

Feature	Chromosome	Plasmid A
Size (bp)	4,610,415	265,602
G + C content (%)	55.91	48.33
Total genes	4425	505
Protein-coding genes	4193	505
rRNA	25	0
tRNA	82	0
Repeat genes	200	0

**Table 2:** Substrate specificity of HylEP0006.

Polysaccharide Species	Relative Enzyme Activity (%)
Hyaluronic acid	100.00 ± 0.71
Chondroitin sulfate	6.33 ± 1.57
Heparin	0.88 ± 0.15
Sodium alginate	0.43 ± 0.21
Chitosan	ND

Note: ND indicates that no enzyme activity is detected. Values represent the mean of three replicates ± SD.

**Table 3:** Effects of metal ions on HylEP0006.

Metal Ions	Relative Enzyme Activity (%)		Metal Ions	Relative Enzyme Activity (%)	
	1 mmol/L	10 mmol/L		1 mmol/L	10 mmol/L
Control	100.00 ± 2.51	100.00 ± 6.50	Ba^2+^	92.06 ± 7.04	71.16 ± 2.54
Ca^2+^	117.52 ± 1.80	143.43 ± 4.11	Zn^2+^	48.42 ± 0.26	12.48 ± 2.38
Mg^2+^	94.54 ± 3.15	152.22 ± 7.40	K^+^	97.36 ± 2.57	74.96 ± 1.82
Li^+^	105.52 ± 0.77	65.93 ± 2.54	Al^3+^	7.39 ± 1.80	9.86 ± 7.40
Cu^2+^	38.93 ± 0.58	ND	Fe^3+^	75.96 ± 1.67	ND

Note: ND indicates that no enzyme activity is detected. Values represent the mean of three replicates ± SD.

**Table 4:** The properties of hyaluronidases from different species.

Source	Molecular Mass	Substrate Spectrum	Optimal Temperature (°C)	Optimal pH	pH Stability	Assay Method	Specific Activity (U/mg)
*E. asburiae* CGJ001 (This study)	87.88 kDa	HA	40	7	6–9	Reducing sugar method (DNS termination)	9.50 × 10^5^
*Bacillus* sp. A50 [21]	123 kDa	HA, CS	44	6.5	5–6	Turbidimetric method	1.02 × 10^6^
*Bacillus niacin* JAM F8 [16]	120 kDa	HA, CS	45	6	6–11	Ultraviolet method	136.7
*Brevibacterium halotolerans* DC1 [22]	41 kDa	HA, CS, DS, dermatan	37	7	5–9	Turbidimetric method	26.37
*Arthrobacter globiformis* A152 [23]	73.7 kDa	HA, CS	42	6	5–7	Ultraviolet method	297.2
*Streptococcus pyogenes bacteriophage* H4489A [24]	40 kDa	HA	37	5.5	4–7	Elson–Morgan-like method	9.62
*Paenibacillus aquistagni* SH-7-A [25]	110 kDa	HA	40	6	5–7	Ultraviolet method	1.18 × 10^4^
*Bacillus* sp. CQMU-D [26]	126.2 kDa	HA, CS	40	7	7–10	Ultraviolet method	-
*Thermasporomyces copostie* DSM22891 [27]	90 kDa	HA	70	5.93	6.1–10.9	Ultraviolet method	10.91
*Yersinia* sp. 298 [28]	115.4 kDa	HA, CS	40	7.5	6.0–11.0	Ultraviolet method	11.19
*Escherichia* sp. A99 [29]	86.7 kDa	HA, CS	40	6	5.5–6.6	Ultraviolet method	376.32
*Homo sapiens* [30]	48.3 kDa	HA	-	3.5–4.0	-	Elson–Morgan-like method	6.8
*Bos grunniens* [31]	55 kDa	HA, CS and DS	37	3.8	-	Elson–Morgan-like method	20.4
*Lachesis muta rhombeata* [32]	60 kDa	-	37	6	-	Turbidimetric method	-

Note: The enzyme activity definition of 1 U determined with various methods: Turbidimetric method–the amount of enzyme required to make 1 µmol hyaluronic acid turbidity vanished per minute; DNS termination–the amount of enzyme required to degrade hyaluronic acid to form 1 µg reducing sugar per hour; Elson–Morgan-like method–the amount of enzyme required to releases 1 µmol of the unsaturated oligomers produced per minute; Ultraviolet method–the amount of enzyme required to degrade hyaluronic acid to form 1 µmol of unsaturated double bonds per minute.

## Data Availability

All data analyzed during this study are included in this published article.

## Supplementary Materials

The following supporting information can be downloaded at: https://www.mdpi.com/article/10.3390/md22090399/s1, Figure S1: Analysis of HA4: (a) Structure of HA4; (b) HA4 was analyzed by HPLC using a YMC-Pack Polyamine II column. (c) Analysis of HA4 using electrospray ionization mass spectrometry (ESI-MS); Figure S2: Analysis of HA10: (a) Structure of HA10; (b) HA10 was analyzed by HPLC using a YMC-Pack Polyamine II column. (c) Analysis of HA10 using ESI-MS.
