# Supplementary material for: Identification and Characterization of a Highly Active Hyaluronan Lyase from Enterobacter asburiae
Source: Mar Drugs. 2024 Aug 31;22(9):399. doi: 10.3390/md22090399 (PMC11432990; doi:10.3390/md22090399)
Supplement: Supplementary file 1 [file marinedrugs-22-00399-s001.zip › marinedrugs-3161409-supplementary.pdf]

Supplementary Data

Identification and Characterization of a Highly Active Hyaluronan Lyase from *Enterobacter asburiae*

1. Methods

To obtain small-molecule hyaluronic acid oligosaccharides of different molecular weights, a solution containing 0.2% (w/v) HA was digested with 500 U/mL purified enzyme for 4 h at 40℃. Next, the samples were heated at 100°C for 10 min to inactivate them, then centrifuged at 12000 rpm for 10 min and filtered (using a cellulose acetate membrane with a pore size of 0.22 μm) for use. Anionic Q-Sepharose Fast Flow was used to purify hyaluronic acid oligosaccharides. The specific steps are as follows: First, equilibration (150-200 mL) with 25 mM Tris HCl (pH 8.0) at a flow rate of 8.0 mL/min, sample injection volume of 5 mL, followed by elution with 300 mL of 25 mM Tris HCl solution. After that, 100 mL each was eluted with 25 mM Tris HCl, 0.05, 0.1 and 0.2 M sodium chloride solution in turn, and finally, the column was cleaned with 1 M sodium chloride solution. Each 10 mL was collected as a tube, and ultraviolet detection was performed at a wavelength of 232 nm. The collected samples were subsequently desalted by Bio-P2 to obtain pure products.

2. Results

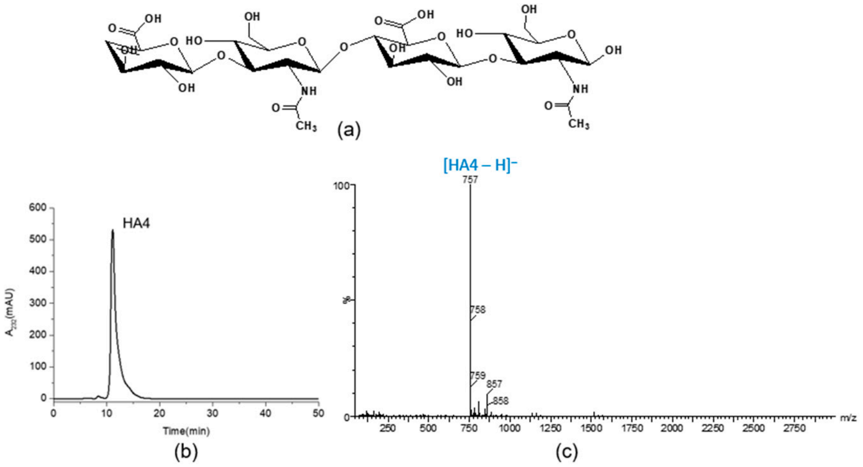

Figure S1. Analysis of HA4. (**a**) Structures of HA4; (**b**) HA4 was analyzed by HPLC using a YMC-Pack Polyamine II column. (**c**) Analysis of HA4 using electrospray ionization mass spectrometry (ESI-MS).

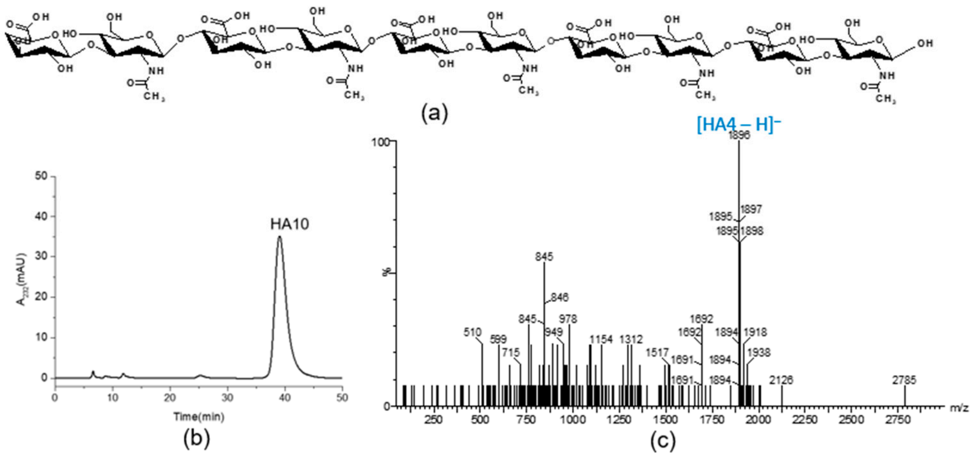

Figure S2. Analysis of HA10. (**a**) Structures of HA10; (**b**) HA10 was analyzed by HPLC using a YMC-Pack Polyamine II column. (**c**) Analysis of HA10 using electrospray ionization mass spectrometry (ESI-MS).
